# Surgical treatment of first branchial cleft anomalies using retrograde facial nerve dissection technique

**DOI:** 10.1007/s00383-025-06122-7

**Published:** 2025-07-22

**Authors:** Ji Won Kim, Moo Kyun Park, Myung-Whan Suh, Soon-Hyun Ahn, Seong Keun Kwon, Jungirl Seok, Yoon Kyung Jeon, Seongyeon Jung, Eun-Jae Chung

**Affiliations:** 1 Department of Otorhinolaryngology-Head and Neck Surgery, Chungnam National University Sejong Hospital, Sejong, Republic of Korea; 2 Department of Otorhinolaryngology-Head and Neck Surgery, Seoul National University Hospital, 101 Daehak-ro, Jongno-gu, Seoul, 03080 Republic of Korea; 3 Department of Pathology, Seoul National University Hospital, Seoul National University College of Medicine, Seoul, Republic of Korea

**Keywords:** First branchial cleft anomalies, Surgical treatment, Facial nerve, Retrograde dissection, Children

## Abstract

**Purpose:**

First branchial cleft anomalies (FBCAs) are infrequent congenital malformations. In FBCAs removal surgery, due to the previous infection history and the anatomical proximity of the FBCAs tract to the facial nerve, postoperative recurrence and facial paralysis are not uncommon. This study aimed to assess the clinical feasibility and outcomes of FBCAs resection using the retrograde facial nerve dissection technique.

**Methods:**

This retrospective study included 19 patients (mean age, 6.3 ± 4.4 years) who underwent FBCAs excision via retrograde facial nerve dissection between 2017 and 2023. Data on demographics, operative details, histopathology, postoperative complications, and follow-up survey were reviewed.

**Results:**

Preoperative infection history was present in 94.7% of patients; 42.1% had prior incision and drainage and 15.8% had previous excision attempts. Complete resection was achieved in all cases without facial nerve palsy. No recurrence was observed during the follow-up periods (median, 23.9 ± 9.8 months). Postoperative pain and paresthesia showed clinical improvement, while cosmetic satisfaction was relatively limited.

**Conclusion:**

In FBCAs patients, the close proximity of the facial nerve and the adhesion between the tract and facial nerve pose significant challenges. Using retrograde facial nerve dissection is believed to enable complete removal and reduce postoperative facial nerve paralysis.

## Introduction

First branchial cleft anomalies (FBCAs) are infrequent congenital malformations, accounting for less than 8% of branchial cleft anomalies [1]. They occur due to the incomplete fusion of the cleft between the first branchial arches and the second branchial arches [1, 2]. The external auditory canal (EAC) and facial nerve originate from the first cleft and second branchial arches, respectively, with parotid gland development occurring upon the obliteration of these arches. FBCAs arise from the persistence of epithelial elements at the site of fusion of the inferior portion of the cleft. These residual epithelial structures can manifest as a cyst, sinus, or complete fistula [3]. Consequently, this embryological process often positions FBCAs near the EAC, parotid gland, and facial nerve, especially the main trunk of the facial nerve [4, 5]. FBCAs are typically identified due to recurrent infections; however, they are often misdiagnosed and undertreated with incision and drainage (I&D) or incomplete resection [5, 6]. Surgical resection is the main treatment for FBCAs; however, the close proximity of the FBCAs tract to the facial nerve and the severe adhesion of the surgical field caused by previous infection and I&D history increase the complexity of a complete resection and raise the risk of facial paralysis [7, 8]. Considering the aforementioned characteristics of FBCAs, the conventional method of facial nerve dissection, which initially identifies the facial nerve trunk, is performed in an unclear surgical plane. Consequently, there exists a potential for unintentionally leaving a residual tract or causing postoperative facial nerve paralysis. To compensate for this, we used retrograde facial nerve dissection as an alternative surgical technique to identify the whole facial nerve branch, including the facial nerve trunk. The aim of this study is to evaluate the demographic and clinicopathological characteristics of patients with FBCAs and to assess postoperative outcomes, including facial nerve palsy, recurrence, and patient satisfaction scores, in individuals who underwent FBCAs’ resection surgery using the retrograde facial nerve dissection technique.

## Methods

### Study design

This study was conducted with signed informed consent under the approval and guidance of the Institutional Review Board of Seoul National University Hospital (SNUH-2504-084-1630). A total of 19 patients were diagnosed with FBCAs based on preoperative clinical presentations and imaging studies and subsequently underwent complete surgical excision using the retrograde facial nerve dissection technique performed by a single otolaryngologist at a single institution from March 2017 to June 2023. Clinical demographic information, operation details, postoperative complications, and histopathologic findings were documented and reviewed. Postoperative assessments for each patient included pain, paresthesia at the operation site, and cosmetic satisfaction scores at 1, 6, and 12 months, respectively. Postoperative pain was evaluated using a visual analog scale (VAS) ranging from 0 (no pain) to 10 (most severe). Postoperative paresthesia at the operation site was scored on a 3-point scale: 0 (none), 1 (mild), and 2 (moderate to severe). Similarly, postoperative cosmetic satisfaction was scored on a 4-point scale: 0 (dissatisfied), 1 (acceptable), 2 (satisfied), and 3 (extremely satisfied).

### Surgical procedures

**Fig. 1:**
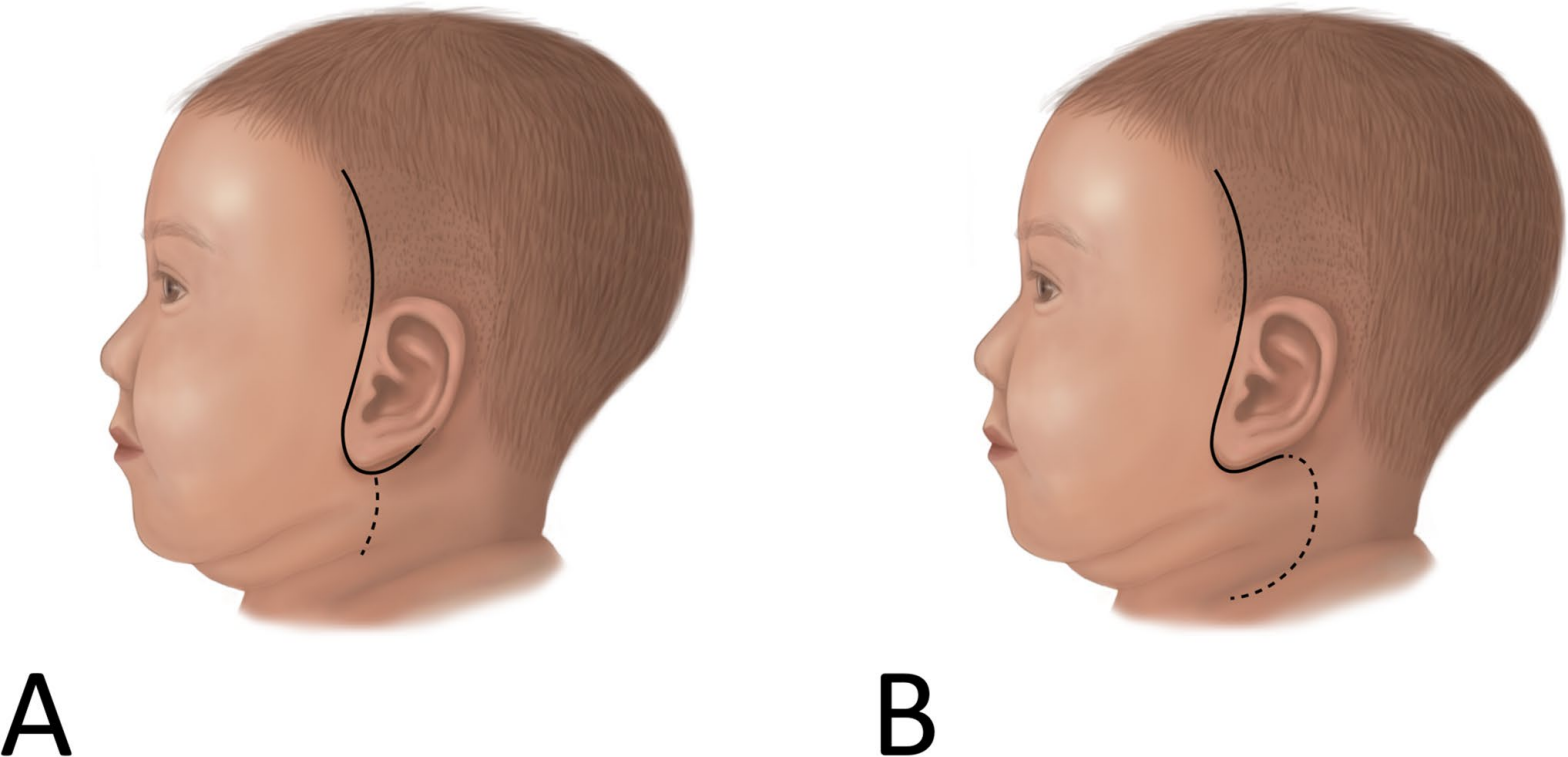
Surgical incision of left FBCAs’ resection. Based on the preauricular incision, the upper temporal extension was made to facilitate the identification of the peripheral facial nerve outside the parotid gland and the lower extension was made along the ear lobule to improve the cosmetic result. **A** Additional incisions (dotted line) may be used in the form of a vertical limb. **B** Additional incision (dotted line) descends toward the neck in a lazy S-shape

**Fig. 2:**
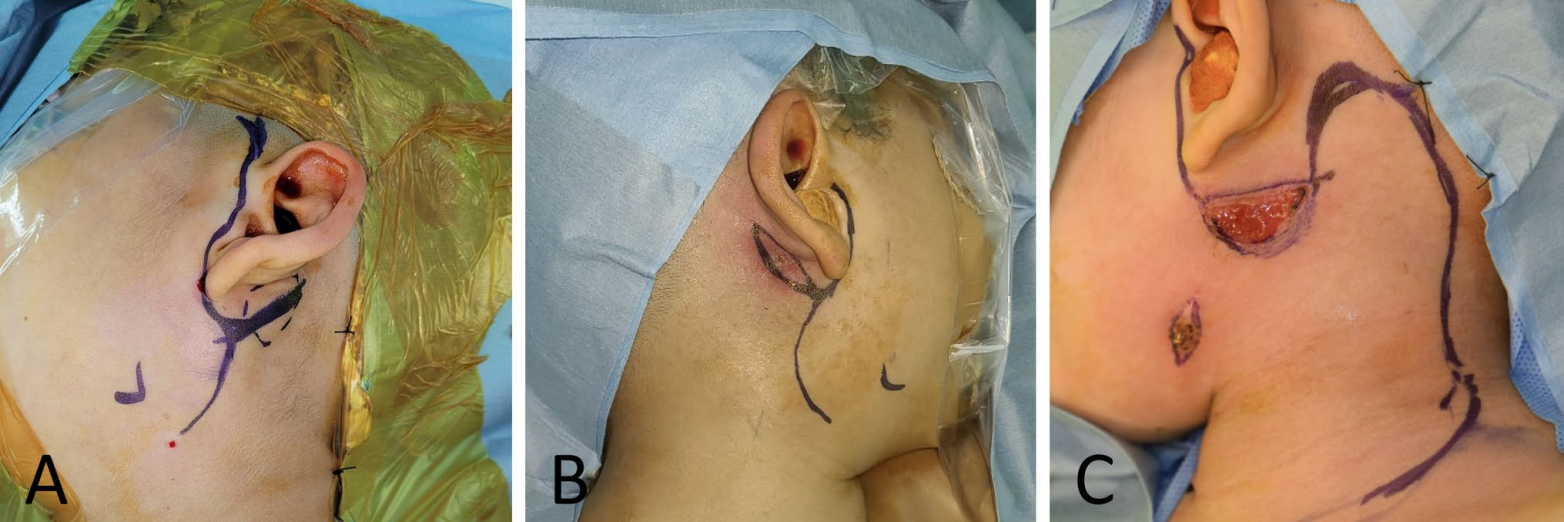
Variable surgical incision depending on the location of the lesion in different patients. If removal of infected skin is required, an elliptical incision around the skin lesion can be made

Intraoperative 4-channel facial nerve monitoring was used in all 19 patients under general anesthesia. For surgical incisions, the surgeon made slight modifications to the traditional parotidectomy incision. The surgical incision was designed as a preauricular incision with an upper temporal extension to facilitate the identification of the terminal branches of the facial nerve as they extend outside the parotid gland, particularly along its anterior and superior borders. The lower extension was made along the ear lobule to improve the cosmetic result. If necessary, a vertical limb or lazy S-shape was added based on the location of the lesion (Fig. 1). If there is a skin defect that requires removal, an elliptical incision is made around the defect (Fig. 2).

After the skin flap is sufficiently elevated, a retrograde facial nerve dissection is performed from the anterior aspect of the lesion rather than the traditional approach of finding the trunk of the facial nerve. The rationale for this is that the fistula tract is often close to the facial nerve, especially the main trunk of the facial nerve [4, 5]. In most cases, the tissue around the lesion and fistula tract is not intact due to a history of previous infection, so it is easier to find and expose the facial nerve from a distance from the lesion, where the tissue is relatively fresh because of less previous inflammatory response.

In retrograde facial nerve dissection, the main branches of the facial nerve that are typically identified are the zygomatic branch, the buccal branch, and the marginal mandibular branch [9, 10]. We usually identify each branch in the following ways. First, the zygomatic branch can be found at the point where the line connecting the incisura to the lateral can thus on the lesioned side meet the zygomaticus major and zygomatic arch on the ipsilateral side. Second, the buccal branch can be found at the point where the line connecting the incisura to the philtrum on the lesioned side meets the margin of the parotid gland on the ipsilateral side. At last, the marginal mandibular branch can be found around the mandibular angle. Following these nerves, a retrograde dissection is performed, starting from the area surrounding the lesion and encompassing the lesion itself, as well as the entire parotid gland. This extensive dissection includes the trunk of the facial nerve and ensures a comprehensive clearance around the lesion and parotid gland.

**Fig. 3:**
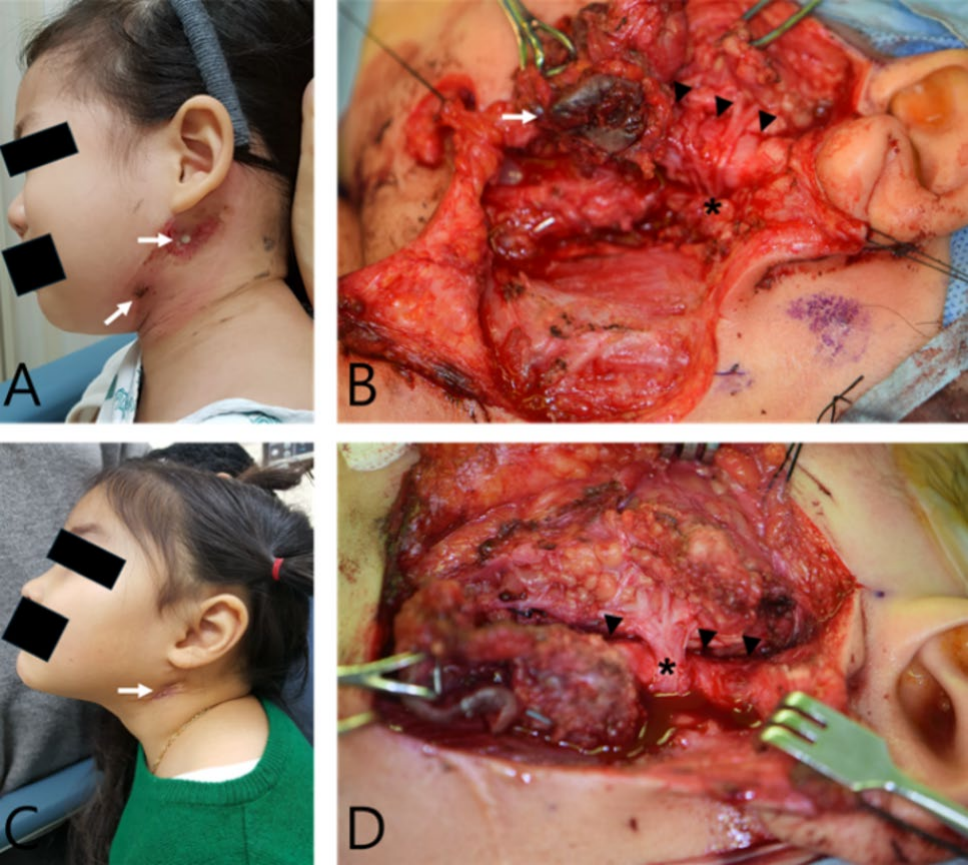
Intraoperative surgical field view of the patients with FBCAs. **A** Preoperative photograph of a 3-year-old female patient with skin defects (white arrow). **B** Intraoperative surgical field view of the same patient, showing the skin defects being excised (white arrow) and the FBCAs tract (black arrow head) passing under the facial nerve trunk (asterisk). **C** Preoperative photograph of a 5-year-old female patient. A skin defect (white arrow) is observed (white arrow). **D** Intraoperative surgical field view of the same patient, showing the FBCAs tract (black arrow head) passing under the facial nerve trunk (asterisk)

**Fig. 4:**
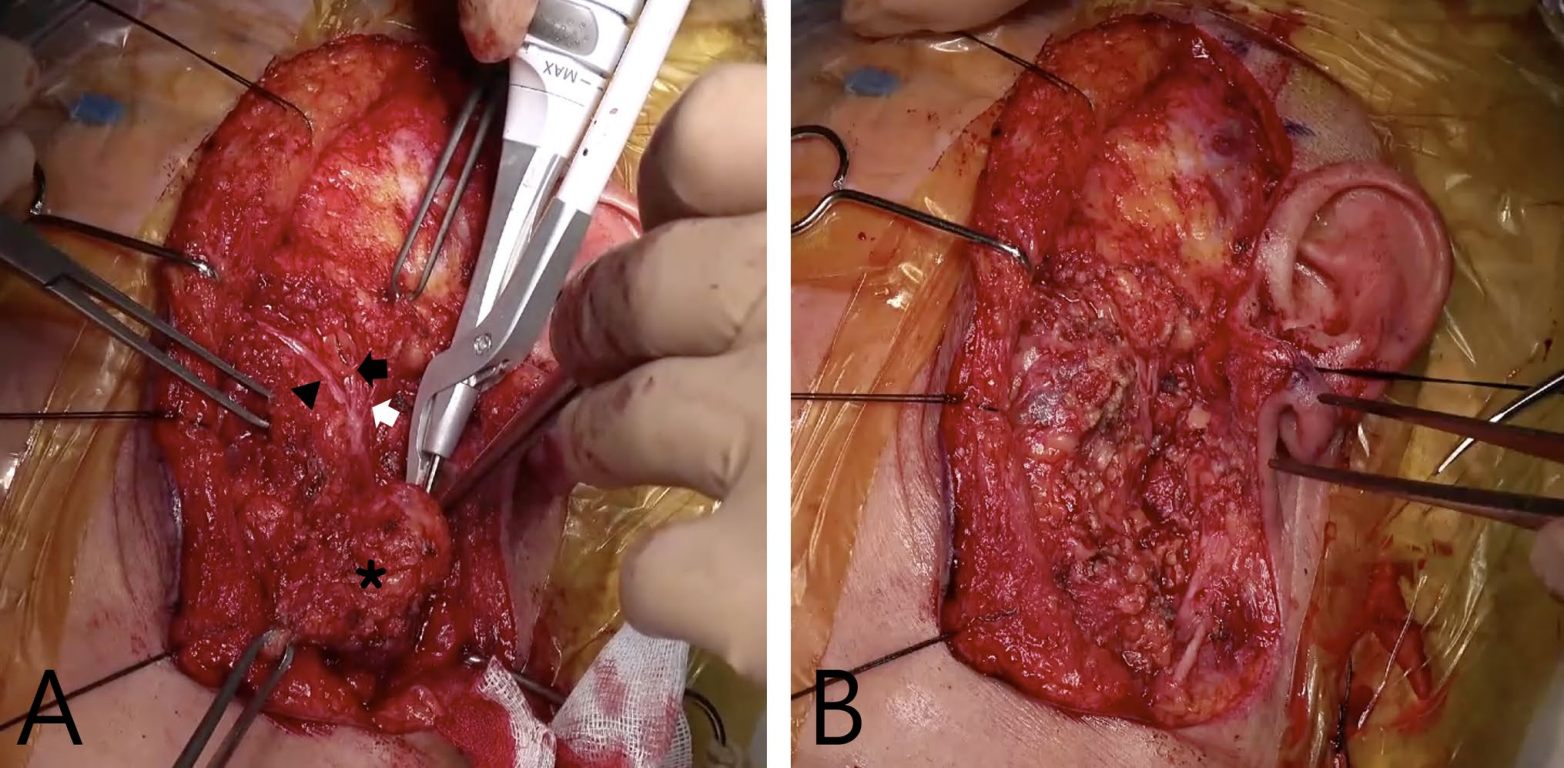
Intraoperative surgical field view of left FBCA resection surgery. **A** The surgical field demonstrates the identification of peripheral branches of the facial nerve, including the zygomatic branch innervating the zygomaticus major muscle (black arrowhead), the zygomatic branch innervating the orbicularis oculi muscle (black arrow), and the temporal branch (white arrow), through peripheral exploration and retrograde dissection. The left first branchial cleft cyst (asterisk) is shown during excision. **B** The surgical field after complete excision of the lesion, including the tract, with complete preservation of the peripheral branches of the facial nerve

After the surgical field is fully exposed, the lesion is resected and the fistula tract is followed and completely removed with careful attention to the facial nerve (Figs. 3, 4). To prevent recurrence, the cartilage of the EAC wall should be removed, including the overlying skin if necessary. Defects of the EAC are typically repaired through primary closure; however, in some cases, reconstruction using the temporalis fascia is required.

## Results

The study cohort included a total of 19 patients (16 males and 3 females; mean age, 6.3 ± 4.4 years, range 2–20 years) and each had a single unilateral lesion. 10 (52.6%) presented on the left side and 9 (47.4%) on the right, representing an almost equal distribution between the two sides. Out of the total 19 patients, 18 (94.7%) had a previous infection history before undergoing surgery, while only 1 (5.3%) did not have a history of infection. Among patients with a previous infection history, 84.2% (16 of 19) had a recurrent infection history, and 57.9% (11 of 19) had a persistent infection history. Additionally, there were 12 (63.2%) patients with a history of previous hospitalization treatment. Furthermore, 8 (42.1%) patients had a history of previous I&D, and 3 (15.8%) patients had a history of previous surgical removal. The demographic and clinicopathological characteristics of the patients are presented in Table 1.

**Table 1:** Patients’ demographic features

Variables	No. (%) of patients
Age, years	
Mean ± SD (range)	6.3 ± 4.4 (2–20)
Sex	
Male/female	16 (84.2)/3 (15.8)
History of recurrent infection	
Presence/absence	16 (84.2)/3 (15.8)
History of I&D	
Presence/absence	8 (42.1)/11 (57.9)
History of surgical excision	
Presence/absence	3 (15.8)/16 (84.2)

*SD* standard deviation, *I&D* incision and drainage

All 19 patients underwent FBCAs resection surgery using retrograde facial nerve dissection. Among 19 patients, 8 (42.1%) had findings of infection at the time of surgery. The mean operation time was 167.9 ± 64.0 (range 60–290) min. The mean length of the resected lesions was 2.2 ± 1.1 (range 0.5–4.5) cm. In histopathologic evaluations, all cases were consistent with FBCAs.

The surgical removal of the FBCAs using the retrograde facial nerve dissection technique was successful for all 19 patients, with no postoperative facial nerve palsy (0%, 0 of 19). And also, there were no postoperative recurrences (0%, 0 of 19) detected during the follow-up period (median, 23.9 ± 9.8 months, range 13–53 months).

**Fig. 5:**
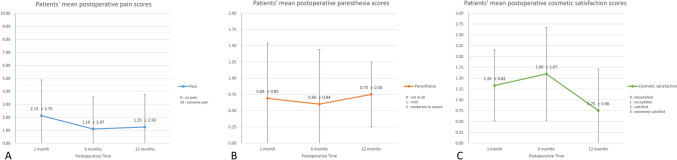
Changes over time in patients’ average scores of symptoms and cosmetic satisfaction. **A** Patients’ mean postoperative pain scores. **B** Patients’ mean postoperative paresthesia scores. **C** Patients’ mean postoperative cosmetic satisfaction scores

Meanwhile, based on surveys conducted during the follow-up period, patients’ mean postoperative pain scores were 2.1 ± 2.7, 1.1 ± 2.5, and 1.3 ± 2.5 (at 1, 6, and 12 months postoperatively, respectively) based on a VAS score (range 0–10). The mean paresthesia scores at 1, 6, and 12 months postoperatively were 0.7 ± 0.9, 0.6 ± 0.8, and 0.8 ± 0.5, respectively, indicating that patients experienced paresthesia ranging from none to mild. The mean cosmetic satisfaction scores at 1, 6, and 12 months postoperatively were 1.3 ± 0.8, 1.6 ± 1.1, and 0.8 ± 1.0, respectively, suggesting that patients rated their cosmetic outcomes as approximately acceptable. The changes over time in the average scores of symptoms and cosmetic satisfaction for patients are described in Fig. 5.

## Discussion

FBCAs are uncommon congenital malformations, accounting for less than 8% of all branchial cleft anomalies [11]. FBCAs typically emerge due to the incomplete fusion of the cleft between the first and second branchial arches, manifesting as a fistula, cyst, or sinus often associated with the external auditory canal, or alternatively, as a neck opening that appears swollen or exhibits signs of inflammation [1, 6, 8, 12].

The accurate preoperative diagnosis of FBCAs is challenging. Several studies have reported that the mean period between the onset of symptoms of FBCAs and the administration of appropriate treatment is between 3.5 and 4 years [12, 13]. The diagnosis of FBCA is based on symptoms, and imaging studies are helpful in making the diagnosis. However, there are other diseases that have similar symptoms and imaging findings to FBCAs. Harnsberger et al. reported that peri-parotid inflammatory mass is sometimes misdiagnosed when CT scans are used to diagnose FBCAs, and Chen et al. reported that the accurate diagnostic rate of enhanced CT and US was 75% and 55.6%, respectively, in their institution [6, 14].

Surgical intervention serves as the primary treatment for FBCAs [7]. When performing surgery, it is important to consider the complete removal of the FBCAs, including the tract, to prevent recurrence, and preservation of the facial nerve to prevent postoperative facial nerve paralysis.

However, several characteristics of FBCAs make it difficult to achieve these goals. As previously discussed, this embryological development often results in the tract of FBCAs locating near the main trunk of the facial nerve [4, 5]. In addition, most FBCAs patients have a history of previous infections. In two studies with relatively large patient populations, the proportion of patients with a history of previous infection was 60.6% by Yang et al. and 92% by Chen et al. [5, 6]. Also, over 50–75% of the patients had been misdiagnosed at other institutions and inadequately treated, requiring I&D or incomplete resection. Due to these characteristics, the facial nerve became adhesive to the tract of FBCAs, resulting in remnant tract or postoperative facial nerve paralysis.

As a result, postoperative complications, such as facial paralysis and lesion recurrence, have been reported in several studies. Chen et al. and Souza et al. reported the rate of postoperative recurrence and facial nerve paralysis in their studies [2, 6]. The postoperative recurrence rates were 3.5% and 12.5%, respectively, and the rates of postoperative facial nerve paralysis, including transient facial paralysis, were 11% and 25%, respectively.

To preserve the facial nerve, it is important to fully expose the facial nerve, which has been emphasized in the other studies [12, 15]. There are two ways to expose the facial nerve. The two methods are antegrade facial nerve dissection, which is the traditional method of identifying the trunk of the facial nerve first, and retrograde facial nerve dissection, which identifies each branch of the facial nerve and dissects it toward the trunk of the facial nerve. In general, it is reported that there is no difference in surgical outcome between the antegrade and retrograde methods of facial nerve dissection on benign parotid lesion excision [16, 17].

However, in the case of FBCAs resection, as aforementioned, the trunk of the facial nerve is located near the main trunk of the facial nerve, and most patients with FBCAs have a history of previous infection and I&D. These make it difficult to perform an antegrade facial nerve dissection, in which the main surgical field is heavily inflamed and has adhesions, making it difficult to preserve the facial nerve while completely dissecting the tract.

Therefore, we used a retrograde facial nerve dissection, safely identified the facial nerve from the periphery and proceeded to dissect toward the trunk, preserving the facial nerve completely, and then performed a complete resection of the entire lesion including the tract.

To facilitate retrograde dissection, the authors designed the surgical incision as a preauricular incision with an upper temporal extension. This approach enabled easier identification of the terminal branches of the facial nerve as they extend outside the parotid gland, particularly along its anterior and superior borders. Furthermore, unlike conventional incisions, this design allowed for the convenient access to the temporalis fascia, which is advantageous for reconstructing EAC defects that may occur during the removal of the tract opening.

We consider that with retrograde facial nerve dissection, the complications of postoperative recurrence and postoperative facial nerve paralysis in FBCAs surgery can be significantly reduced compared to the conventional method using the antegrade facial nerve, which is supported by the postoperative recurrence rate (0 of 19, 0%) and the incidence of postoperative facial nerve palsy (0 of 19, 0%) at our institution. Furthermore, within the postoperative follow-up survey of the entire patient cohort, both postoperative pain and paresthesia scores at the site of the operation exhibited comparatively favorable advancements. In comparison, the patient’s postoperative cosmetic satisfaction scores were relatively low, which may be related to the fact that the wound of FBCAs is primarily on inflamed skin, and the preoperative skin infected lesions are removed together, or the extension of the incision is inevitably long depending on the extent of the lesion.

Despite these promising findings, several methodological limitations must be considered. First, the retrospective design of this study and its small sample size (*n* = 19) limit the precision of effect estimates. Second, the absence of a control group undergoing the antegrade dissection method and the lack of formal comparative statistical analyses preclude definitive conclusions regarding the relative superiority of the retrograde approach. Third, the median follow-up duration of approximately 24 months may be insufficient to detect late recurrences or long-term complications. Finally, selection bias is possible, as most patients had prior infections or I&D procedures, likely reflecting referral patterns to our institution as a tertiary care institution.

Given these methodological limitations, further prospective studies with larger sample sizes, longer follow-up periods, and adequately matched control groups undergoing antegrade dissection are necessary to systematically compare postoperative complication and recurrence rates between the two surgical approaches. Despite these limitations, our study highlights the potential clinical importance of retrograde facial nerve dissection as an alternative surgical strategy to the conventional antegrade approach in the management of FBCAs. By addressing specific clinical challenges, such as anatomical proximity to the facial nerve trunk and significant local inflammation secondary to prior infections, this technique may help reduce both postoperative complications and recurrence rates. Thus, our findings provide a valuable foundation for future systematic investigations aimed at optimizing surgical methods and improving overall clinical outcomes in patients with FBCAs.

## Conclusion

In FBCAs’ patients, the close proximity of the facial nerve, especially its main trunk, to the tract of FBCAs and the significant adhesion between the FBCAs tract and the facial nerve due to the previous infection and I&D history present a significant surgical challenge. These make it difficult to achieve the dual objectives of preserving facial nerve function and completely removing the lesion, including the tract. Compared to the conventional antegrade facial nerve dissection, using retrograde facial nerve dissection may facilitate both complete lesion removal and reduce the likelihood of postoperative facial nerve paralysis.

## Data Availability

No datasets were generated or analyzed during the current study.
